# Lower Salinomycin Concentration Increases Apoptotic Detachment in High-Density Cancer Cells

**DOI:** 10.3390/ijms131013169

**Published:** 2012-10-12

**Authors:** Ju-Hwa Kim, Tae Young Kim, Hyung Sik Kim, Suntaek Hong, Sungpil Yoon

**Affiliations:** 1 Research Institute, National Cancer Center, Ilsan-gu, Goyang-si, Gyeonggi-do 410-769, Korea; E-Mail: jua@ncc.re.kr; 2 Lee Gil Ya Cancer and Diabetes Institute, Gachon University, Incheon 406-840, Korea; E-Mail: sthong@gachon.ac.kr; 3 Department of Systems Biology, Yonsei University, Seoul 120-749, Korea; E-Mail: taenggury@naver.com; 4 College of Pharmacy, Pusan National University, Busan 609-390, Korea; E-Mail: hkims@pusan.ac.kr

**Keywords:** salinomycin, apoptosis, high cell density, spaces among cells, cellular detachment, lower concentration

## Abstract

The present study identified a novel salinomycin (Sal) sensitization mechanism in cancer. We tested whether Sal reduced proliferation in a high-density population by counting attached cell numbers after Sal treatment. Sal reduced proliferation in high-density cell populations. Longer exposure to Sal further reduced proliferation. Sal concentrations of 0.1 and 5 μM had similar sensitization effects, suggesting that Sal toxicity was minimal with longer exposure to a high-density cell population. The results suggest that Sal can be applied at a relatively low concentration for a longer time to overcome drug-resistant solid tumors. The 0.5 μM Sal treatment resulted in fewer attached cells than that of the 5 μM Sal treatment with a longer exposure. The lower Sal concentration mainly increased the number of easily detachable cells on the surface. In particular, 0.5 μM Sal increased cellular detachment of newly produced daughter cells. The easily-detachable cells were undergoing apoptosis. It seems that the 0.5 μM Sal treatment also increased cellular toxicity. These novel findings may contribute to the development of Sal-based therapy for patients with drug-resistant cancer or a high-density solid tumor.

## 1. Introduction

Salinomycin (Sal) is an anionic and weakly acidic compound. Sal is produced by *Streptomyces albus* as a 751 Da monocarboxylic polyether, and acts on both cytoplasmic and mitochondrial membranes as an ionopore with strict selectivity for alkali ions and a great preference for potassium. Sal can facilitate bidirectional ion flux through lipid membranes by passive diffusion, in which Sal forms lipid-soluble complexes with cations. Sal exhibits antimicrobial activity and is widely used as an antiprotozoal agent against parasites responsible for the poultry disease coccidiosis, for example in chickens, pigs, as well as ruminants. It is used for improving nutrient absorption and feeding efficiencies for the treated creatures [1–4].

Sal was originally used to eliminate bacteria, fungi and parasites [1,4]. More recently, the compound has been used to inhibit the growth of tumor stem cells and chemoresistant cancer cells [5–17]. Sal also functions as an efflux pump P-glycoprotein (P-gp) inhibitor [18–20]. Sal is considered to be a potential anti-cancer drug for cancer chemoprevention; Sal sensitizes cancer cells to the effects of doxorubicin (DOX), etoposide (ETO), radiation and anti-mitotic drugs, resulting in apoptosis by causing DNA damage and reducing p21 protein levels through increased proteasomal activity [19,21,22]. A more complete understanding of the mechanism governing Sal sensitization could facilitate the therapeutic use of Sal in patients with cancer.

Increased cell density in cell culture model systems causes resistance to anti-cancer drugs. Similarly, *in vivo* high-density solid tumors exhibit resistance to anti-cancer drugs [23]. In the present study, we investigated the capability of Sal to sensitize a high-density culture. Sal sensitization was compared between low density and high-density cultures, and using different concentrations of Sal. In addition, Sal sensitization was also compared between days one and two to observe the effect of treatment time.

The effects of Sal were facilitated by a number of sensitization mechanisms including inhibition of ionophores, increased DNA damage, and prevention of P-gp pumping. The current data demonstrate another Sal sensitization mechanism evident in high-density culture. This novel finding of a Sal sensitization mechanism could facilitate the therapeutic use of Sal in patients with cancer.

## 2. Results and Discussion

### 2.1. Attached Cells in High Density Culture are more Effectively Reduced by Longer Sal Exposure

High density confluent cultured cells are resistant to anti-cancer drugs, likely precluding the rapid growth of solid tumors *in vivo* [23]. We tested the relationship of Sal sensitization and increased cancer cell density. Hs578T breast cancer cells were seeded in 60 mm-diameter dishes at initial cell numbers of 2 × 10^5^ (low density) or 4 × 10^5^ (high density). The number of attached cells was enumerated after Sal treatment. The Sal sensitization effect was compared with different concentrations of Sal (5, 2, 1, 0.5, and 0.1 μM). Sal sensitization was also compared between days one and two to observe the effect of treatment time.

**Figure 1:**
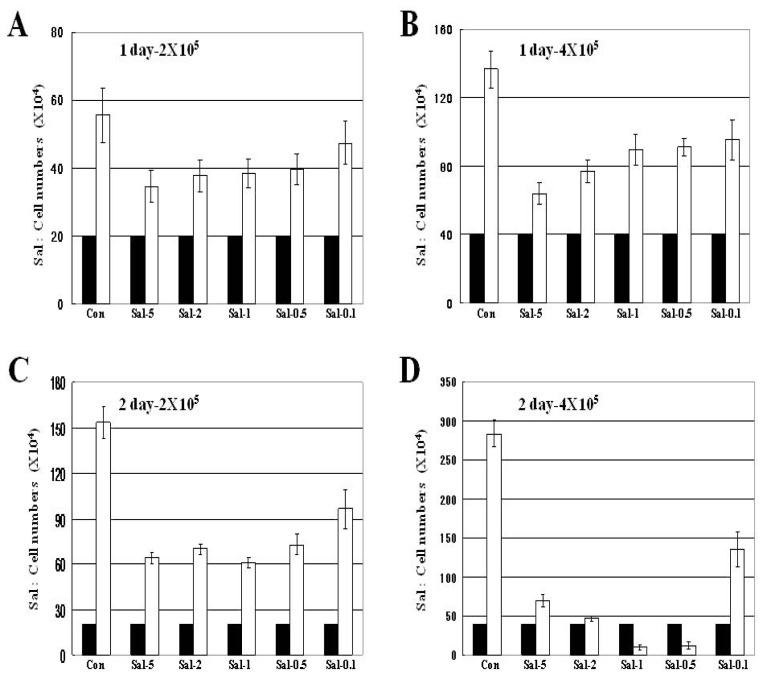
Attached cells in high-density culture are more effectively reduced by longer exposure to Sal. (**A**–**D**) Hs578T cells with 2 × 10^5^ or 4 × 10^5^ were plated on 60 mm-diameter dishes. The cells were then incubated for 1 or 2 days with 5 μM Sal (Sal-5), 2 μM Sal (Sal-2), 1 μM Sal (Sal-1), 0.5 μM Sal (Sal-0.5), 0.1 μM Sal (Sal-0.1), or dimethylsulfoxide (DMSO; Con). White bars indicate cell numbers after 1 day or 2 days of Sal treatment. Black bars indicate the initial cell numbers (2 × 10^5^ or 4 × 10^5^) before drug treatments. Attached cells were counted after one or two days.

In Figure 1A,B, the black bars indicate initial cell numbers and white bars are the increased cell numbers after one day. Control cells increased about three-fold, whereas Sal-treated cells increased about two-fold (Figure 1A). Comparison of low density and high-density cultures revealed a similar increase in cell numbers, suggesting the cell density independence of Sal sensitization. Both concentrations of Sal had a similar sensitization effect, suggesting that Sal sensitization was also very effective at the lower concentration. Cell numbers were compared between low and high cell density after two days of Sal treatment to observe the effect during a longer Sal exposure. The cell numbers were much less in cultures seeded with the 4 × 10^5^ cells than those in the 2 × 10^5^ cultures, when compared with the increased cell numbers in the control (Figure 1C,D). This finding suggested that cell numbers were markedly inhibited by Sal in a higher cell density population. The results were confirmed using cultures seeded with 2 × 10^5^, 4 × 10^5^, and 6 × 10^5^ cells, indicating that a more dense culture resulted in more reduced cell numbers than that of lower density cultures (Figure S1A–C). A temporal comparison of Sal sensitization (one day *vs*. two days) using high-density cells (Figure 1B,D), showed that the longer Sal exposure resulted in increased sensitization. The above results show a statistically significant correlation between the percentage of reduced cell numbers, and most Sal concentrations, when compared between days 1 and 2. Taken together, the results indicate that Sal increased sensitization in a high-density cell population with a longer exposure and a lower concentration.

### 2.2. Treatment with 0.5 μM Sal Reduces the Number of Attached Cells Better than 5 μM Sal in High Density Cultures with Longer Exposure

An experiment analyzed the effects of longer Sal exposure in high-density cultures (Figure 1D). When Sal treatments of 5, 2, 1, 0.5, and 0.1 μM were compared in high density cultures at two days (Figure 1D and Figure S1B,C), it was revealed that 0.5 μM Sal resulted in fewer cell numbers than 1, 2, and 5 μM Sal treatments (Figure 1D), indicating that 0.5 μM Sal was the optimal concentration for the sensitization effect in high density cultures at 2 days. The above results show a statistically significance in percentage of reduced cell numbers by 0.5 μM Sal, when compared to 2 or 5 μM Sal. The results also suggested Sal sensitization was independent of Sal concentration in high-density culture during longer exposures.

### 2.3. Treatment with 0.5 μM Sal Increases Easily-Detachable Cells in High Density Culture

**Figure 2:**
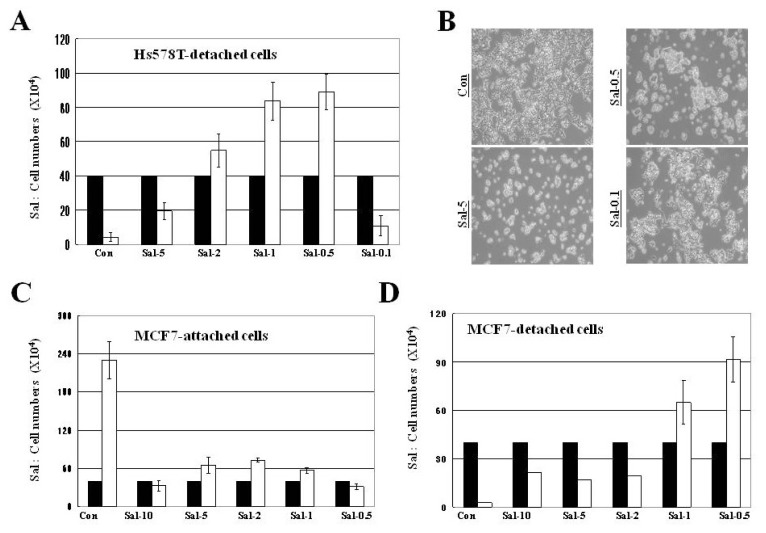
Treatment with 0.5 μM Sal markedly increases easily-detachable cells in high density culture. (**A**) Hs578T cells (4 × 10^5^) were plated on 60 mm-diameter dishes. The cells were then incubated for two days with 5 μM Sal (Sal-5), 2 μM Sal (Sal-2), 1 μM Sal (Sal-1), 0.5 μM Sal (Sal-0.5), 0.1 μM Sal (Sal-0.1), or DMSO (Con). White bars indicate cell numbers after two days of Sal treatment. Black bars indicate the initial cell numbers (4 × 10^5^) before drug treatments. Supernatant detached cells were counted after two days. The average total numbers of cells (attached and detached) are approximately 28.8 × 10^5^ for Con, 8.9 × 10^5^ for Sal-5, 10.2 × 10^5^ for Sal-2, 9.4 × 10^5^ for Sal-1, 10.2 × 10^5^ for Sal-0.5, and 14.6 × 10^5^ for Sal-0.1. (**B**) Hs578T cells (4 × 10^5^) were plated on 60 mm-diameter dishes. The cells were then incubated for two days with 5 μM Sal (Sal-5), 0.5 μM Sal (Sal-0.5), 0.1 μM Sal (Sal-0.1), or DMSO (Con). The cells were observed after 2 days using an inverted microscope with a 5× objective lens. (**C**, **D**) MCF7 cells (4 × 10^5^) were plated on 60 mm-diameter dishes. The cells were then incubated for three days with 10 μM Sal (Sal-10), 5 μM Sal (Sal-5), 2 μM Sal (Sal-2), 1 μM Sal (Sal-1), 0.5 μM Sal (Sal-0.5), or DMSO (Con). Attached (**C**) or supernatant detached (**D**) cells were counted after three days.

We sought to identify why treating high-density cells with 0.5 μM Sal-treatment had a better sensitization effect and fewer cell numbers than the higher concentration treatments. To approach this, we tested whether cellular detachment by 0.5 μM Sal treatment occurred in the high density culture by enumerating cells in the supernatant before trypsinization, which included both detached cells in the medium and washed cells in phosphate buffered saline (PBS). Treatment with 0.5 μM Sal primarily increased the number of detached cells, when compared with the control, 5 μM Sal, or 0.1 μM Sal (Figure 2A). The above results show a statistically significant reduction in cell numbers by 0.5 μM Sal, when compared with 0.1 or 5 μM Sal. When the numbers of attached and detached cells were ascertained in the various Sal concentrations (Figure 1D *vs*. Figure 2A), the superior sensitization effect (*i.e*., fewer cells) produced by the 0.5 μM Sal treatment in high-density cultures was shown to have resulted from increases in easily detachable cells from the surface.

Additionally, when we compared cell numbers among the initial cells (4 × 10^5^; black bars in Figure 1D,2A), attached cells, and detached cells in the 0.5 μM Sal treatments (Figure 1D *vs*. Figure 2A), an increase in total cells to about 9 × 10^5^ was evident after two days, suggesting that proliferation occurred in the 0.5 μM Sal treatment. However, the number of attached cells was much lower than the number of detached cells. The attached cell numbers were even smaller than the initial cell numbers, suggesting that the 0.5 μM Sal treatment increased the detachment of both newly divided daughter cells and parent cells. We assume that the low concentration of Sal is insufficient for inhibiting the growth of parent cells in the short-term. Considering that 0.5 μM Sal needs more time for its accumulation inside cells, we assume that 0.5 μM Sal could not inhibit parent cell growth in the short-term. It means that cellular accumulation of a certain amount of Sal is required for inhibiting growth of parent cells. However, we suppose that 0.5 μM Sal could sufficiently sensitize the newly divided daughter cells.

### 2.4. Treatment with 0.5 μM Sal Increases Proliferation in High Density Culture, but not Differentiation for Attachment

Sal-treated cells were examined microscopically. As shown in Figure 2B, the number of cells increased in the 0.5 μM and 0.1 μM Sal-treated cell cultures compared to those in the 5 μM Sal-treated cultures. The morphology of the 0.1 μM Sal-treated cells was similar to that of the control cells. But, the newly divided daughter cells in the 0.5 μM Sal treatment appeared rounder. The 0.5 μM Sal-treated cells were undifferentiated when compared with the control and 0.1 μM Sal-treated cells. Considering that the newly divided daughter cells did not differentiate for attachment, we assumed that the 0.5 μM Sal-treated cells were easily detached from the surface. The results favored the suggestion that 0.5 μM Sal increased both cellular proliferation and detachment. We observed similar results in the MCF7 cancer cell line (Figure 2C,D), indicating that the mechanism of 0.5 μM Sal sensitization was conserved in various cancer types.

**Figure 3:**
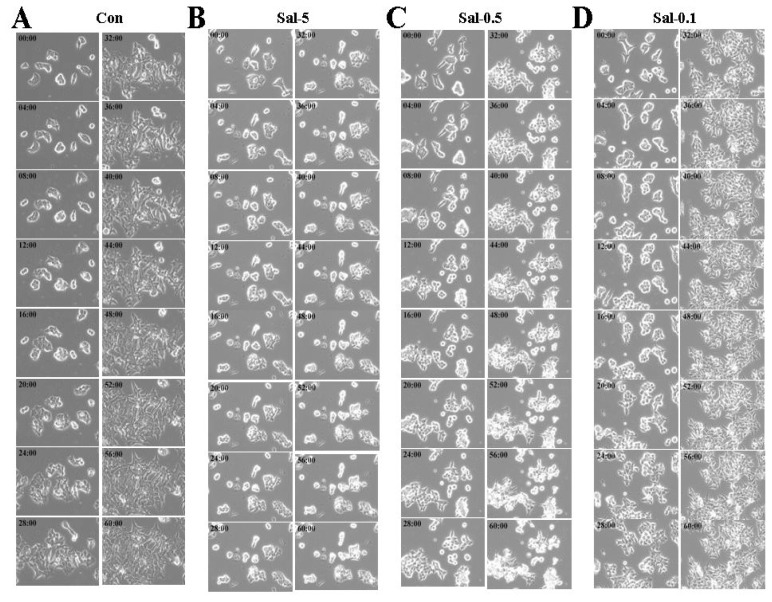
Treatment with 0.5 μM Sal increases proliferation in high-density culture, but not differentiation for attachment. (**A**–**D**) Hs578T cells (4 × 10^5^) were plated on 6-well plates. The cells were then incubated for three days with 5 μM Sal (Sal-5), 0.5 μM Sal (Sal-0.5), 0.1 μM Sal (Sal-0.1), or DMSO (Con). Live cell imaging was performed by taking pictures every 20 min with a 20× objective lens to estimate growth, division, and detachment. The numbers in each picture indicate time after drugs treatments. Pictures are shown with a 4 h interval.

We observed live cell growth images taken every 20 min during three days. The representative results shown in Figure 3A–D confirmed the microscopic observations in Figure 2B. The increased detachment due to the 0.5 μM Sal treatment took more than two days (Figure 3C), which agreed with the observation of increased detachment in 0.5 μM Sal-treated cells after two days (longer exposure) compared to one day (shorter exposure) (Figure 1A–D). This finding was consistent with the suggestion that the increase in the number of detached cells came from newly divided daughter cells. The control and 0.1 μM Sal-treated cells also displayed increased cell numbers in separate groups, and each group was close and combined together (Figure 3A,D). However, cells were barely combined with each other in the 0.5 μM Sal-treated cells (Figure 3C). It seemed that Sal also inhibited the combining process and then increased the number of cellular detachments. We assumed that non-combining process and non-differentiation among groups contributed to the increased cellular detachment of the 0.5 μM Sal-treatment at 2 days.

### 2.5. Treatment with 0.5 μM Sal Markedly Increases Apoptotic Cells in High Density Culture

**Figure 4:**
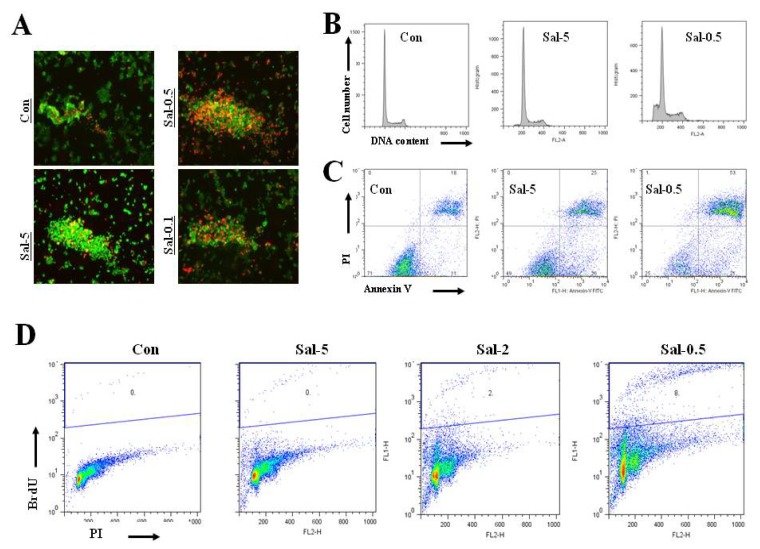
Treatment with 0.5 μM Sal markedly increases apoptotic cells in high density culture. (**A**) Hs578T cells (4 × 10^5^) were plated on 96-well plates. The cells were then incubated for two days with 5 μM Sal (Sal-5), 0.5 μM Sal (Sal-0.5), 0.1 μM Sal (Sal-0.1), or DMSO (Con). After two days, cell viability (live/dead assay) was then measured. The cells were observed using an inverted fluorescence microscope with a 5× objective lens. (**B**) Hs578T cells (4 × 10^5^) were plated on 60 mm-diameter dishes. The cells were then incubated for two days with 5 μM Sal (Sal-5), 0.5 μM Sal (Sal-0.5), 0.1 μM Sal (Sal-0.1), or DMSO (Con). FACS analysis was performed after two days. (**C**, **D**) Hs578T cells 4×10^5^ were plated on 60 mm-diameter dishes. The cells were then incubated for two days with 5 μM Sal (Sal-5), 2 μM Sal (Sal-2), 0.5 μM Sal (Sal-0.5), or DMSO (Con). Annexin V analysis (**C**) or TUNEL assay (**D**) was performed after two days.

Although we expected that the detached cells underwent a form of apoptosis known as anoikis [24], we experimentally tested whether 0.5 μM Sal increased the number of apoptotic cells. Treatment with 0.5 μM Sal resulted in more dead cells than those in the 5 μM and 0.1 μM Sal treatments (Figure 4A). We confirmed the results with repeated experiments, which showed similar patterns. Fluorescence activated cell sorting (FACS) analysis for pre-G1 phase, Annexin V analysis, and terminal transferase dUTP nick end labeling (TUNEL) staining revealed that the 0.5 μM Sal treatment increased apoptosis more than the 5 μM Sal treatment (Figure 4B–D). These results were consistent with the idea that the detached cells from the 0.5 μM Sal-treatment were in anoikis. We conclude that the 0.5 μM Sal treatment increased the proportion of cells in apoptosis through increased detachment.

We also tested whether ETO, a well-known anti-cancer drug [21], had similar effects. No sensitization effect for detachment was evident with a lower concentration of ETO in high-density cultures with longer exposures (Figure S2A–F), suggesting that the 0.5 μM Sal-sensitization mechanism is different from etoposide.

### 2.6. Treatment with 0.5 μM Sal may Increase Cellular Toxicity in High Density Cultures

Sal is a P-gp inhibitor [18–20]. Sal has been shown to be a substrate of P-gp *in vivo* [25]. We wanted to determine whether the P-gp inhibitory role correlated with Sal treatment concentration. Rhodamine123 (Rho) was used to test whether Sal increased the inhibition of P-gp substrate efflux. Rho is a well-known substrate used to measure P-gp inhibition [19]. In this experiment, cellular accumulation of green fluorescence was indicative of Rho intracellular concentration. As shown in Figure S3, Sal concentrations ranging from 2 to 10 μM increased Rho staining, suggesting that Sal contributes to P-gp inhibition at high Sal concentrations.

In contrast, Sal concentrations < 1 μM markedly reduced Rho staining compared to that in the control (Figure S3). In particular, 0.5 μM Sal-treated cells showed the lowest Rho staining, which was only one-third that of the control. Reduced Rho staining has been used to measure drug toxicity by mitochondrial damage [26,27]. Therefore, the elevated reduction in Rho staining seems to be indicative of increased cellular damage. In order to confirm increased toxicity in the 0.5 μM Sal-treated cells, further analysis is required. The present results support the suggestion that the increased toxicity of 0.5 μM Sal may contribute to the increased detachment of newly divided daughter cells. We also assumed that undifferentiated newly divided cells in the 0.5 μM Sal treatment were positively correlated with increased toxicity.

### 2.7. Space among Cells Contributes to Increased Detachment in 0.5 μM Sal Treatment

Lastly, we were interested in whether 0.5 μM Sal in a low-density population mimicked the high-density culture. As shown in Figure S4A–C, 1 μM or 0.5 μM Sal treatment of low density cultures increased detachment, apoptosis and toxicity more than observed using 5 μM or 2 μM Sal treatment. These results indicated that the increased detachment caused by 0.5 μM Sal generally occurred in both low and high density populations, and also demonstrated that the detachment occurred faster in the higher density population. We confirmed the results with repeated experiments, which showed similar patterns.

We were interested in determining why high cell density facilitated increased detachment compared to low cell density. As shown in Figures 2B and 3A–D, an increased number of daughter cells caused by 0.5 μM Sal reduced cellular spaces in high density cultures. The increased number of daughter cells resulting from the 0.5 μM Sal treatment narrowed the spaces among the cell groups, whereas more spaces were evident in the low-density culture. We assumed that the Sal treatment also reduced cellular detachment. Therefore, it could be concluded that the reduced cellular spaces due to the increased number of daughter cells positively facilitated increased cellular detachment by 0.5 μM Sal in the high-density culture.

## 3. Discussion

Our results first focused on the increased cell numbers observed in various Sal concentrations. When we observed attached cell numbers, we found that proliferation was greatly inhibited by Sal in the higher density population. Highly dense cells are more resistant to anti-cancer drugs [23]. However, presently, Sal sensitization was independent of increased cell density. Longer exposure to Sal resulted in more sensitization, which may indicate the value of longer Sal exposure in treating drug-resistant solid tumors. We also found that 5 μM Sal had a similar sensitization effect as 0.1 μM Sal-treated cells, for both longer exposures and higher density cultures. Toxicity associated with relative high Sal concentrations may be harmful to normal cells [28]. Our results indicate that Sal toxicity is minimal with longer exposures, particularly in a high-density population. These results also suggest that the sensitization effects of Sal for clinical applications can be achieved with relatively low concentrations of Sal that avoid toxicity and harm to normal cells.

Presently, Sal sensitization was independent of Sal concentration in high-density cultures during longer exposures. Treatment with 0.5 μM Sal markedly increased easily detachable cells on surfaces of high-density cultures. The 0.5 μM Sal treatment showed increased proliferation in the high-density culture, but not differentiation for attachment. Considering that newly divided daughter cells do not differentiate for attachment, we assumed that they were easily detached from the surface. It is of interest to further investigate the proteins involved in the regulation of detachment.

In addition, the 0.5 μM Sal treatment increased detachment of both newly divided daughter cells and parent cells, and the number of attached cells was smaller than the detached cell numbers. The attached cell numbers were less than that initial cell numbers, suggesting that 0.5 μM Sal treatment increases the detachment of both newly divided daughter cells and parent cells. We conclude that Sal inhibits the stable attachment of both newly divided daughter and parents cells on surfaces.

We also observed increased detachment by the 0.5 μM Sal treatment in lower density cultures, although the number of detached cells was much smaller than that of high-density cultures. These results suggest that the increased detachment by 0.5 μM Sal generally occurs in both low and high-density populations. It also indicates that faster detachment can be achieved in higher density populations using 0.5 μM Sal. When we identified the reason for the difference between high and low density cultures, we observed an increased number of daughter cells narrowed down the spaces among cell groups. But, more spaces among cell groups existed in low-density cultures. We assume that increased space among cell groups reduces the number of cellular detachments.

Therefore, we conclude that the reduced cellular spaces by the increased number of daughter cells positively facilitate increased cellular detachment in high-density cultures.

## 4. Experimental Section

### 4.1. Reagents

Sal and ETO were purchased from Sigma-Aldrich (St. Louis, MO, USA). Rhodamine123 (Rho) was purchased from Santa Cruz Biotechnology (Santa Cruz, CA, USA).

### 4.2. Cell Culturing

Previously described human cancer cell lines [19,21,22,29,30] were used. Hs578T breast cancer cells were obtained from the Korean Cell Line Bank (Seoul, Korea). MCF7 breast cells were obtained from the American Type Culture Collection (Manassas, VA, USA). All cell lines were cultured in DMEM or RPMI 1640 containing 10% fetal bovine serum, 100 U/mL penicillin, and 100 μg/mL streptomycin (WelGENE, Daegu, Korea).

### 4.3. Cell Counting

Briefly, cells grown in 60 mm-diameter dishes were washed with 5 mL of phosphate buffered saline (PBS), dislodged with trypsin, and pelleted by centrifugation for 1 min at 3000 rpm. The pellet was suspended in 500 μL of culture medium. The number of total attached cells was counted using a hematocytometer. Cells in both the supernatant medium and washed PBS were also counted for the detached cells. All experiments were performed independently at least three times with triplicates in each experiment.

### 4.4. Live Cell Imaging

Live cell imaging was performed as described previously [31] to estimate cellular growth, division, and detachment. Briefly, cells were incubated under the same culture conditions and medium. Pictures were taken every 20 min for 72 h; 0.5-s exposures were acquired using a 20× NA0.75 objective LSM500 META confocal microscope (Carl Zeiss, Oberkochen, Germany).

### 4.5. Cell Viability Assay

A Live/Dead assay kit (Invitrogen, Carlsbad, CA, USA) was used according to the manufacturer’s instructions to measure cell viability. Briefly, cells were grown in 96-well plates and treated with the indicated drugs for the prescribed times. The medium was removed from the cells before they were stained with calcein-AM and ethidium homodimer dissolved in PBS. Calcein-AM, a nonfluorescent polymeric dye, is retained by live cells and produces intense green fluorescence through enzymatic (esterase) conversion. The ethidium homodimer enters cells with damaged membranes and binds nucleic acids, thereby producing bright red fluorescence in dead cells. Cells were analyzed under a fluorescence microscope after incubating for 30 min at room temperature. We performed at least three independent tests.

### 4.6. Fluorescence-Activated Cell Sorting (FACS) Analysis

FACS analysis was performed as described previously [19,21,22,29,30]. Cells were grown in 60-mm dishes and treated with the indicated drugs for the prescribed times. The cells were then dislodged by trypsin and pelleted by centrifugation. The pelleted cells were washed thoroughly with PBS, suspended in 75% ethanol for at least 1 h at 4 °C, washed again with PBS, and re-suspended in a cold propidium iodide (PI) staining solution (100 μg/mL RNase A and 50 μg/mL PI in PBS) for 40 min at 37 °C. The stained cells were analyzed for relative DNA content using a FACSCalibur flow cytometry system (BD Bioscience, Franklin Lakes, NJ, USA). We performed at least three independent tests.

### 4.7. Annexin V Analysis

Annexin levels were measured as described previously [19,21,22,29,30]. Annexin V-fluorescein isothiocynate (FITC) staining was performed using a commercial Annexin V-FITC kit (BD Bioscience, San Diego, CA, USA). Briefly, cells were harvested as described for the FACS analysis. Cells (1 × 10^5^) in 100 μL of binding buffer were incubated with 5 μL of Annexin V-FITC and 5 μL of PI (50 μg/mL) for 15 min at RT. The stained cells were analyzed using a FACSCalibur flow cytometry system.

### 4.8. Terminal Transferase dUTP Nick End Labeling (TUNEL) Assay

TUNEL analysis was conducted with FITC-anti-BrdU staining using a commercial APO-BRDU kit (Phoenix Flow Systems, San Diego, CA, USA). Briefly, cells were harvested as described above for the FACS analysis. Cells (1 × 10^6^) were fixed in 1% paraformaldehyde in PBS (pH 7.4), added to 5 mL of 70% ethanol, and stored for 20 h at −20 °C. The cells were harvested by centrifugation, washed in buffer, resuspended in DNA labeling solution consisting of TdT Reaction Buffer, TdT Enzyme, and BrdUTP; and incubated for 60 min at 37 °C. The cells were rinsed with 1.0 mL of rinse buffer, treated with FITC-anti-BrdU antibody solution, and incubated for 30 min at room temperature. The cells were added to 0.5 mL of PI/RNase A staining solution and incubated for 15 min at room temperature. The stained cells were analyzed using a FACSCalibur flow cytometry system.

### 4.9. Cellular Rho Level Tests

These tests were used to determine P-gp inhibition or drug-toxicity using a previously described method [19]. Briefly, cells were placed in six-well plates, treated with indicated drugs, and incubated for 48 h at 37 °C. Cells were then incubated with 1 μg/mL Rho for 1 h 30 min at 37 °C. The medium was removed, and the cells were washed twice with PBS. The stained cells were subsequently examined using an inverted fluorescence microscope. We performed at least three independent tests.

### 4.10. Statistical Analysis

Data are presented as the mean ± standard deviation. The statistical analysis was conducted using Student’s *t*-test and a one-way analysis of variance followed by a multiple-comparison test. Results were considered statistically significant compared to the control when *p* < 0.05.

## 5. Conclusions

Increased cell density in cell culture model systems causes resistance to anti-cancer drugs. Similarly, *in vivo* high-density solid tumors exhibit resistance to anti-cancer drugs. However, we found that Sal effectively reduced attached cell numbers in a high-density culture, particularly when administered for a prolonged time at a relatively low concentration. We also showed that the reduction in attached cell numbers was positively correlated with increased apoptosis.

The present study adds to the list of Sal sensitization mechanisms for newly-divided cancer cells, which is increased cellular detachment and apoptosis in narrowed cellular spaces during high density culture in the case of both low concentrations and longer exposures. Our findings may contribute to the development of Sal-based therapies for patients.

## Supplementary Materials
