# Supplementary material for: Lower Salinomycin Concentration Increases Apoptotic Detachment in High-Density Cancer Cells
Source: Int J Mol Sci. 2012 Oct 12;13(10):13169–82. doi: 10.3390/ijms131013169 (PMC3497319; doi:10.3390/ijms131013169)
Supplement: Supplementary file 1 [file ijms-13-13169-s001.pdf]

Supplementary Information

Figure S1. Reduction of attached cell numbers by Sal positively correlates with increased cell density. (**A**–**C**) Hs578T cells at 2 × 10^5, 4 × 10^5, or 6 × 10^5 were plated on 60 mm-diameter dishes. The cells were then incubated for 2 days with 5 µM Sal (Sal-5), 0.5 µM Sal (Sal-0.5), 0.01 µM Sal (Sal-0.01), or DMSO (Con). White bars indicate cell numbers after 2 days of Sal treatment. Black bars indicate the initial cell numbers (2 × 10^5 or 4 × 10^5) before drug treatments. The cells were counted after 2 days.

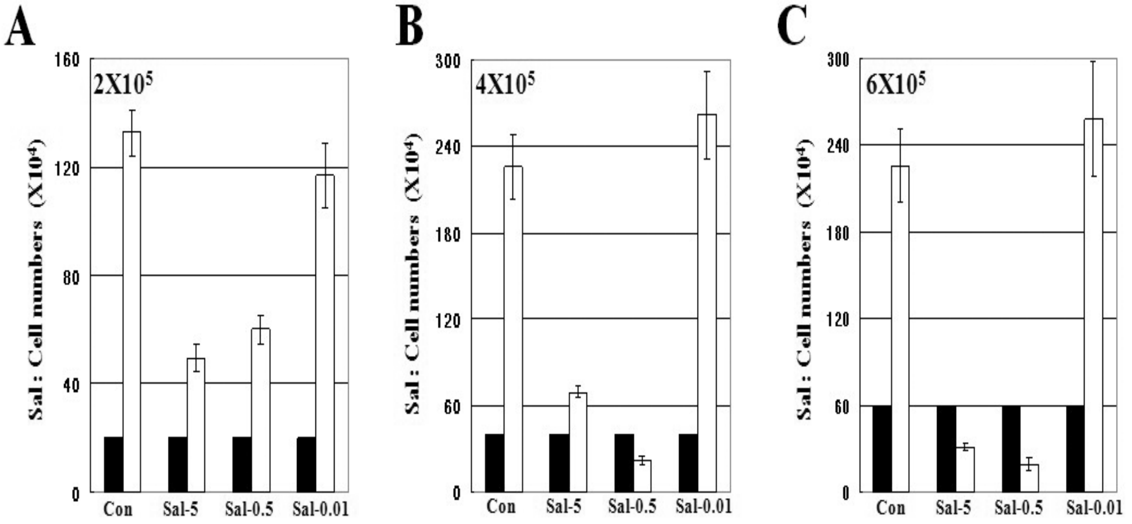

Figure S2. ETO does not increase the sensitization effect for detachment at a lower concentration in high density culture. (**A**) Hs578T cells (4 × 10^5) were plated on 60 mm-diameter dishes. The cells were then incubated for 2 days with 100 µM ETO (ETO-100), 20 µM ETO (ETO-20), or DMSO (Con). After 2 days, the cells were observed using an inverted microscope with a 5X objective lens. (**B**) Hs578T cells (4 × 10^5) were plated on 60 mm-diameter dishes. The cells were incubated for 2 days with 100 µM ETO (ETO-100), 10 µM ETO (ETO-10), or DMSO (Con). White bars indicate cell numbers after 2 days of ETO treatment. Black bars indicate the initial cell numbers (4 × 10^5) before drug treatments. The attached cells were counted after 2 days. (**C**) Hs578T cells (4 × 10^5) were plated on 60 mm-diameter dishes. The cells were incubated for 2 days with 100 µM ETO (ETO-100), 10 µM ETO (ETO-10), or DMSO (Con). White bars indicate cell number after 2 days of ETO treatment. Black bars indicate initial cell numbers (4 × 10^5) before drug treatments. Supernatant detached cells were counted after 2 days. (**D**) Hs578T cells (4 × 10^5) were plated on 96-well plates. The cells were incubated for 2 days with 100 µM ETO (ETO-100), 20 µM ETO (ETO-20), or DMSO (Con). Cell viability (live/dead assay) was measured after 2 days. The cells were observed using an inverted fluorescence microscope with a 5× objective lens. (**E**) Hs578T cells (4 × 10^5) were plated on 60 mm-diameter dishes. The cells were incubated for 2 days with 100 µM ETO (ETO-100), 50 µM ETO (ETO-50), 20 µM ETO (ETO-20), 10 µM ETO (ETO-10), 5 µM ETO (ETO-5), or DMSO (Con). White bars indicate cell numbers after 2 days of ETO treatment. Black bars indicate initial cell number (4 × 10^5) before drug treatments. Detached supernatant cells were counted after 2 days. (**F**) Hs578T cells (4 × 10^5) were

plated on 96-well plates. The cells were incubated for 2 days with 10 μM ETO (ETO-10), 2 μM ETO (ETO-2), or DMSO (Con). Cell viability (live/dead assay) was measured after 2 days. The cells were observed under an inverted fluorescence microscope with a 5× objective lens.

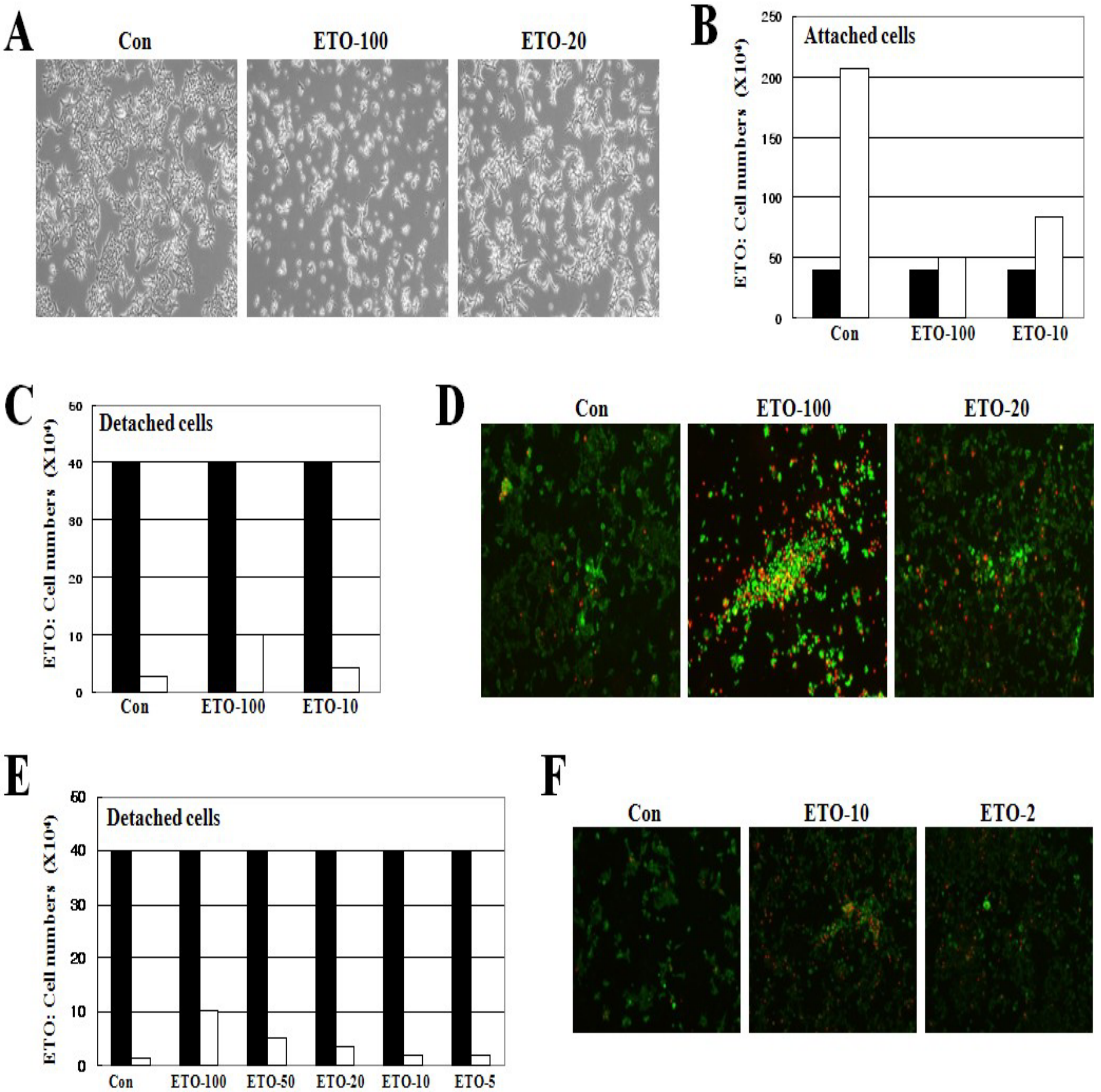

Sup Fig. 2

Figure S3. The 0.5 μM Sal treatment may appreciably increase cellular toxicity in high density cultures. Hs578T cells (4 × 10^5) were plated on 60 mm-diameter dishes. The cells were incubated for 2 days with 10 μM Sal (Sal-10), 5 μM Sal (Sal-5), 2 μM Sal (Sal-2), 1 μM Sal (Sal-1), 0.5 μM Sal (Sal-0.5), 0.1 μM Sal (Sal-0.1), 0.01 μM Sal (Sal-0.01), or DMSO (Con). The cells were then stained with Rho after 2 days. FACS analysis was subsequently performed for the Rho stained cells.

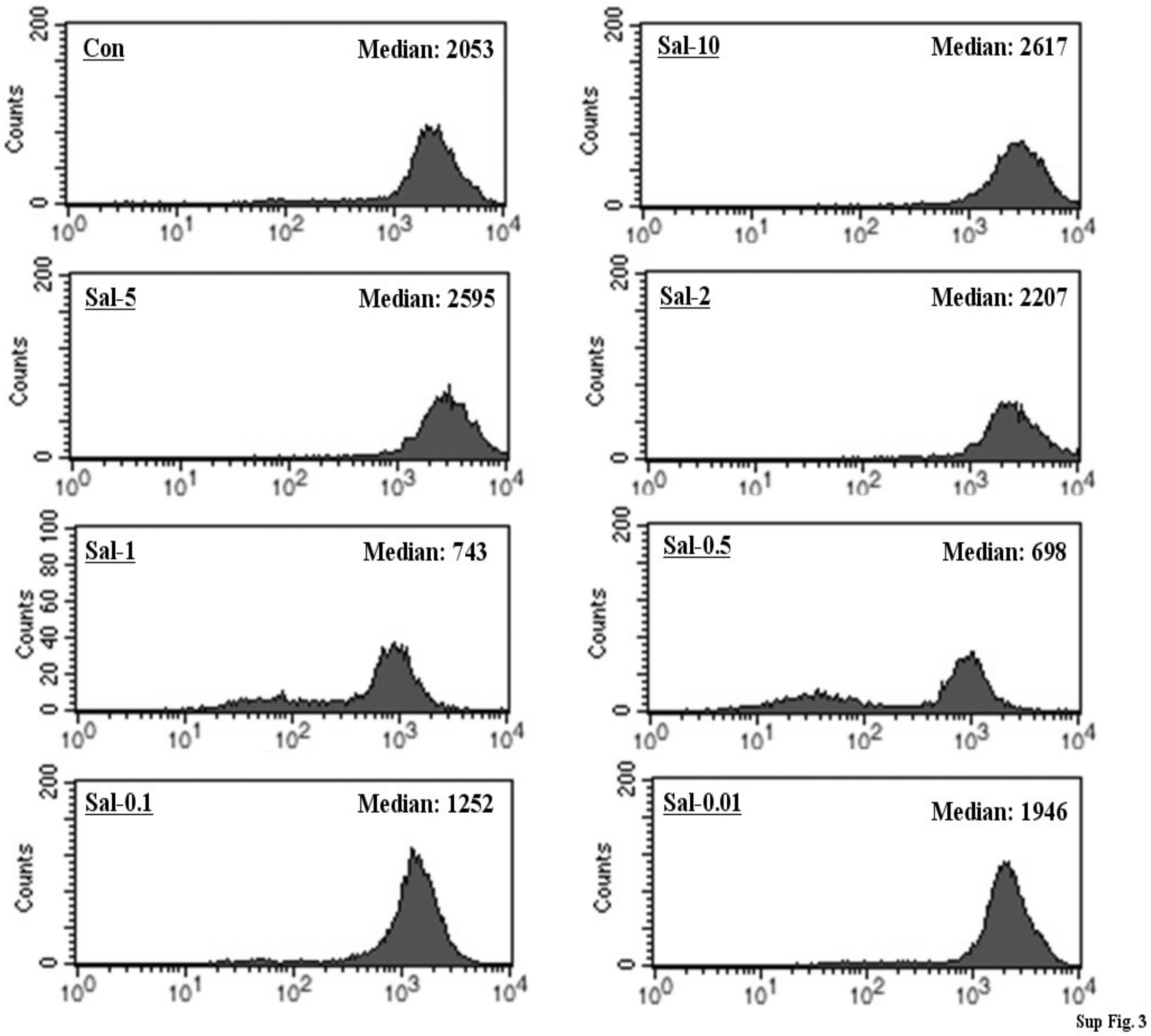

Figure S4. Increased cellular detachment caused by 0.5 μM Sal treatment can be also observed at low cell density. (A) Hs578T cells (2 × 10^5) were plated on 60 mm-diameter dishes. The cells were then incubated for 2 days with 5 μM Sal (Sal-5), 2 μM Sal (Sal-2), 1 μM Sal (Sal-1), 0.5 μM Sal (Sal-0.5), 0.1 μM Sal (Sal-0.1), or DMSO (Con). White bars indicate cell numbers after 2 days of Sal treatment. Black bars indicate the initial cell numbers (2 × 10^5) before drug treatments. Supernatant detached cells were counted after 2 days. The average total numbers of cells (attached and detached) are approximately 15.5 × 10^5 for Con, 6.8 × 10^5 for Sal-5, 7.7 × 10^5 for Sal-2, 7.7 × 10^5 for Sal-1, 8.5 × 10^5 for Sal-0.5, and 10.0 × 10^5 for Sal-0.1. (B) Hs578T cells (2 × 10^5) were

plated on 96-well plates. The cells were then incubated for 2 days with 5 μM Sal (Sal-5), 1 μM Sal (Sal-1), 0.5 μM Sal (Sal-0.5), 0.1 μM Sal (Sal-0.1), 0.01 μM Sal (Sal-0.01), or DMSO (Con). Cell viability (live/dead assay) was measured after 2 days. The cells were observed using an inverted fluorescence microscope with a 5× objective lens. (C) Hs578T cells (2×10^5) were plated on 60 mm-diameter dishes. The cells were incubated for 2 days with 5 μM Sal (Sal-5), 2 μM Sal (Sal-2), 1 μM Sal (Sal-1), 0.5 μM Sal (Sal-0.5), 0.01 μM Sal (Sal-0.01), or DMSO (Con). The cells were stained with Rho after 2 days. FACS analysis was subsequently performed for the Rho stained cells.

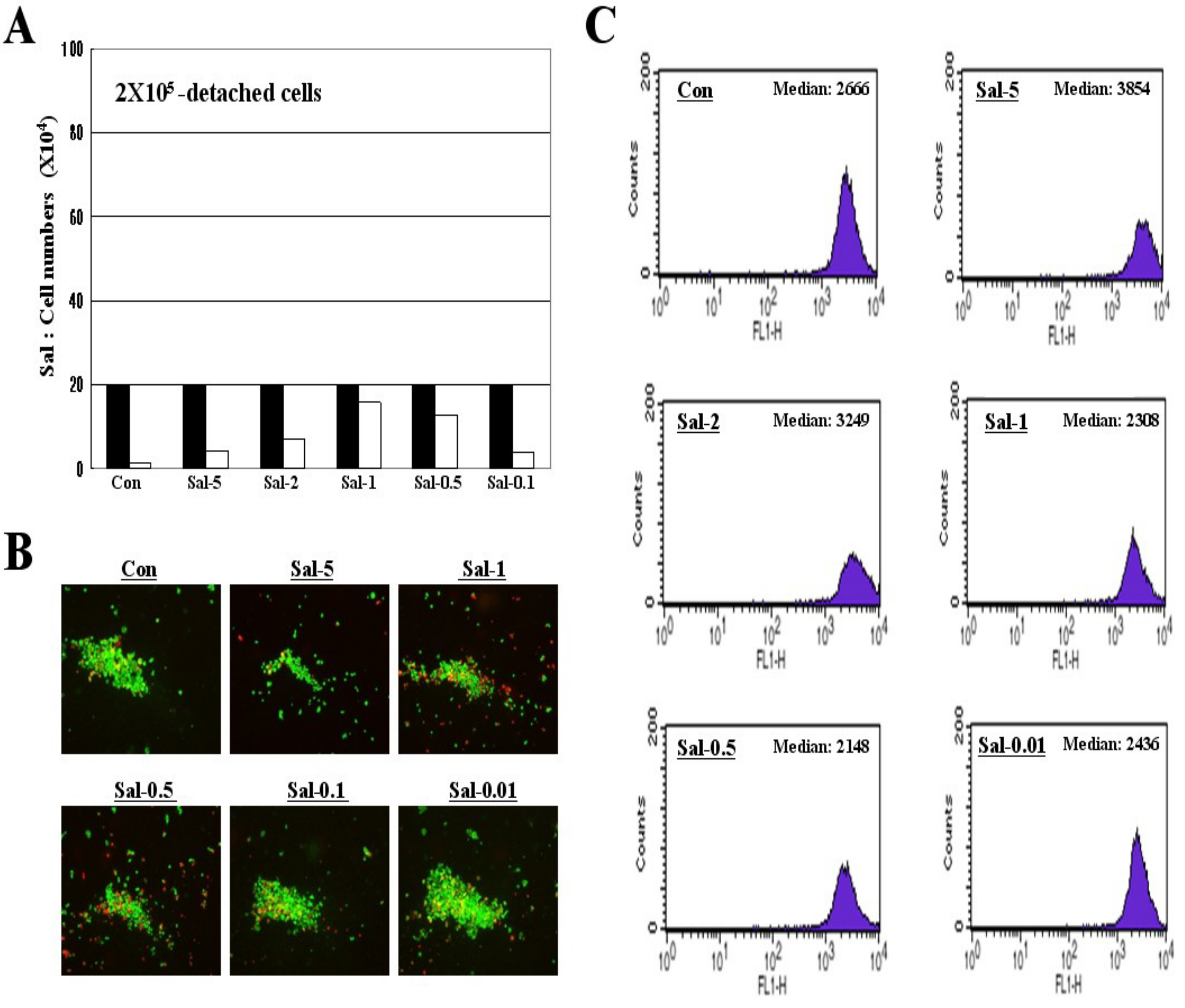

Sup Fig. 4

© 2012 by the authors; licensee MDPI, Basel, Switzerland. This article is an open access article distributed under the terms and conditions of the Creative Commons Attribution license (http://creativecommons.org/licenses/by/3.0/).
